# Only repeatedly elevated IgG4 levels in primary sclerosing cholangitis may distinguish a particular patient phenotype

**DOI:** 10.1186/s12876-024-03343-3

**Published:** 2024-08-05

**Authors:** Sandra Kalthoff, Caroline Wolniak, Philipp Lutz, Christian P. Strassburg, Bettina Langhans, Leona Dold

**Affiliations:** 1 Department of Internal Medicine I, University Hospital of Bonn, Venusberg-Campus 1, D-53127 Bonn, Germany; 2 German Center for Infection Research (DZIF), Partner Site Cologne-Bonn, Bonn, Germany

**Keywords:** PSC, Autoimmune liver disease, Longitudinal sIgG4 measurements, Disease progression

## Abstract

**Background:**

Primary sclerosing cholangitis (PSC) is a chronic liver disease leading to inflammation with scaring and strictures of bile ducts, which can lead to liver cirrhosis. A subtype of PSC characterized by high serum IgG4 (sIgG4) levels has been reported to be associated with poor outcomes, but the exact role and the longitudinal development of sIgG4 levels in PSC progression remains to be clarified. The aim of this study was to investigate if subsequent analysis of sIgG4 levels allows the identification of the PSC phenotype with high sIgG4.

**Methods:**

sIgG4 values were repeatedly analysed in a well-characterized European PSC cohort of 110 individuals. Biochemical parameters, clinical endpoints, death and liver transplantation were compared between PSC subgroups.

**Results:**

12.7% (*n* = 14) of PSC patients showed increased sIgG4 levels (PSC-IgG4). The values normalized in 57.1% (*n* = 8; PSC-IgG4_norm_) during follow-up measurements, whereas the values remained permanently elevated in 42.9% (*n* = 6; PSC-IgG4_const_). Serum values of AP and γGT were significantly higher in PSC-IgG4_const_ compared to PSC-IgG4_norm_ at final blood sampling. Furthermore, mean age at PSC diagnosis was markedly lower in PSC-IgG4_const_ compared to PSC-IgG4_norm_.

**Conclusions:**

This is the first study analyzing longitudinal development of sIgG4 in PSC. Our data indicate that only sequential determination of sIgG4 levels allow to accurately distinguish between the PSC phenotype with high sIgG4 and PSC with low sIgG4.

**Graphical abstract:**

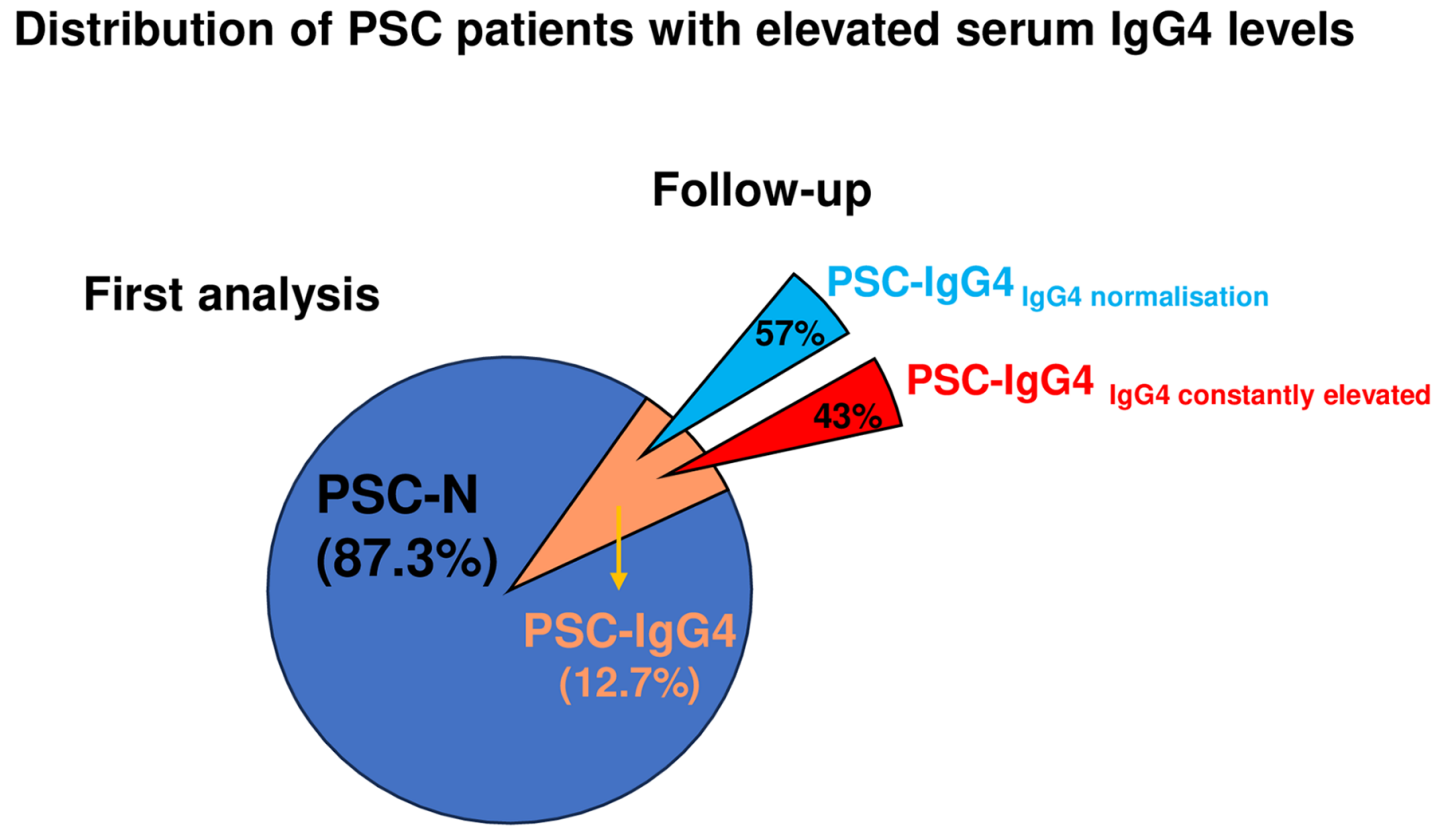

**Supplementary Information:**

The online version contains supplementary material available at 10.1186/s12876-024-03343-3.

## Background

Primary sclerosing cholangitis (PSC) is a chronic liver disease leading to hepatobiliary inflammation, fibrosis, and stricturing of large bile ducts, and in a number of cases also to liver cirrhosis [1]. PSC predominantly affects males and is diagnosed at an early age, most often in or before the 3rd decade of life [2]. 70% of PSC patients also suffer from inflammatory bowel disease (IBD). The risk of cholangiocarcinoma and colorectal cancer is significantly higher than in the general population. Apart from liver transplantation, there is no curative therapeutic option for PSC. The prognosis of untreated PSC ranges between 12 and 21 years of transplant free survival after diagnosis [3]. Immunoglobulin G 4-associated cholangitis (IAC) is a biliary disease with cholangiographic features, which are often indistinguishable from PSC, but is characterized by corticosteroid therapy responsiveness [4]. Therefore, it is important to differentiate IAC from PSC, and determinations of serum IgG4 (sIgG4) are recommended for differential diagnostic reasons in PSC patients. However, differentiating between PSC and IAC can be challenging, because about 10–25% of PSC patients exhibit elevated levels of sIgG4 [5] in the absence of HISORt criteria required for the diagnosis of IAC [6, 7].

PSC can be divided into several subtypes including “classical” large-duct PSC, small-duct PSC, and a PSC/AIH (autoimmune hepatitis) variant-phenotype. An additional subtype of PSC characterized by high sIgG4 has been suggested [5]. In this subtype sIgG4 levels are elevated to levels below the cut-off suggested to support a diagnosis of IAC. Elevated sIgG4 levels in PSC patients have been reported to be associated with poor outcomes in PSC, but the exact role of sIgG4 levels in PSC progression remains to be fully understood [8]. Several studies demonstrated a higher risk of progression to cirrhosis and a decreased time to liver transplantation in PSC patients with high sIgG4 compared to those with serum levels within normal limits [5, 9, 10]. In contrast, a Japanese study failed to find an effect of sIgG4 levels at diagnosis on the prognosis of PSC [11]. Similarly, Benito de Valle and colleagues confirmed that high sIgG4 levels were not associated with an increased risk of liver transplantation, liver-related death or cholangiocarcinoma in a European PSC cohort [12].

The European Association for the Study of the Liver Clinical Practice Guideline (EASL CPG) on PSC suggests the determination of sIgG4 in every adult patient with large-duct sclerosing cholangitis at the time of diagnosis [6]. However, to date there are no data on the follow-up of sIgG4 levels in PSC patients and it is unclear whether a later developing elevation of sIgG4 levels may be missed if measured only at a specific time point. If high levels of sIgG4 are associated with a poorer PSC prognosis, the information regarding the longitudinal development of sIgG4 levels could be clinically relevant. Therefore, the aim of the study was to investigate the time course of sIgG4 levels in a well-characterized European PSC cohort.

## Methods

### Patients

120 PSC patients from the University Hospital in Bonn were included in this retrospective study. We excluded 10 PSC patients with only one measurement of sIgG4. 110 PSC patients were included into the final analysis.

All patients were diagnosed with PSC according to the EASL CPG [6]. None of the patients had a history of pancreatitis. IAC was excluded by sIgG4 levels < 4x UNL, MRCP imaging, and histological analysis of IgG4-positive plasma cells in biliary biopsies derived from ERC and some patients had additional liver biopsy. Patients with diagnosed IgG4-related sclerosing cholangitis and small-duct PSC were excluded from the study, while PSC patients with PSC/AIH variant-phenotype were included. AIH was diagnosed according to the international guidelines [6]. IBD phenotypes were determined according to local expertise and were classified as either ulcerative colitis or Crohn’s disease according to the German Guideline [13]. All patients with a PSC/AIH variant-phenotype received immunosuppressive therapy according to the current guidelines. Therapy was initiated with prednisolone or budesonide and then continued with azathioprine to maintain remission. Immunosuppressive therapy was also administered in some patients to treat IBD. 44 of these patients (40.0%) received either prednisolone, azathioprine, budesonide, vedolizumab, tofacitinib, adalimumab, infliximab, or combinations.

Patient age at PSC diagnosis, gender, date of diagnosis and status of inflammatory bowel disease, biochemical parameters, serum levels of all immunoglobulins (including IgG1, IgG2, IgG3, and IgG4 as well as total IgG), medications, liver stiffness using transient elastography by Fibroscan^®^, malignancies, death or liver transplantation were obtained from our center’s database.

Baseline laboratory parameters were determined at the time of first sIgG4 measurement, and during follow up. All 110 PSC patients had at least two available sIgG4 measurements.

The study was performed in accordance with the Declaration of Helsinki and was approved by the Ethics Committee of the Medical Faculty of the University of Bonn (number 128/23-EP). Informed consent was obtained from each patient.

### Measurement of sIgG4

Serum IgGs were routinely measured in all included patients by immunonephelometry using a Siemens Healthcare Diagnostics assay. Since 2017, sIgG4 were measured by turbidimetry using a commercially available kit (The Binding Site Limited, Birmingham, UK). Due to various changes of the respective normal ranges by the laboratory, ratios of measured sIgG4 in relation to upper limit of normal (ULN) were determined for statistical analysis. PSC patients were classified as PSC-N subgroup if sIgG4 value ratios were equal or lower than 1.0, and as PSC-IgG4 subgroup if one sIgG4 measurement was higher than 1.0, respectively.

### Statistical analysis

Statistics were calculated using GraphPad Prism (version 9.0; GraphPad Prism, San Diego California, USA) and the IBM SPSS Statistics software (version 27; IBM, New York, USA). Datasets were analyzed for normality and tested by unpaired non-parametric Mann Whitney test or Wilcoxon matched-paired signed rank test, respectively. Correlations between sIgG4 ratios and clinical parameters were compared by Spearman rank correlation analyses. *P*-values < 0.05 were regarded to indicate statistical significance.

## Results

### Patient characteristics

In this study, we enrolled 110 patients with PSC, including 58 males and 52 females. None of our patients had small-duct PSC. 79 (71.8%) PSC patients had concomitant IBD (58 ulcerative colitis, 13 Crohn’s disease, 8 indeterminate colitis) and 24 had features of a PSC-AIH variant-syndrome. 19 (17.3%) PSC patients exhibited liver cirrhosis as indicated by histology, transient elastography, or clinical evidence based on typical combinations of laboratory and imaging findings. During the observation time, liver transplantation was performed in three PSC patients (2.7%) and two (1.8%) patients died. Three PSC patients had a history of colorectal cancer and two of biliary malignancy (gallbladder carcinoma). Table 1 summarizes the patients’ characteristics.

**Table 1:** Comparison of demographics and haematological parameters in primary sclerosing cholangitis (PSC) patients

	PSC all (*n* = 110)	PSC-N (*n* = 96)	PSC-IgG4 (*n* = 14)	*p*_1_-value		PSC-IgG4_norm_ (*n* = 8)	PSC-IgG4_const_ (*n* = 6)	*p*_2_-value
Sex, male: female (male %)	58:52 (52.7%)	46:50 (47.9%)	12:2 (85.7%)	0.008		6:2 (75.0%)	6:0 (100%)	n.s.
Age at PSC diagnosis	34.5 (11–75)	35.4 (11–75)	28.6 (12–50)	n.s.		37.0 (16–50)	17.5 (12–21)	0.0067
Observation time (years)	4.4	4.5	4.4	n.s.		3.9	5.0	n.s.
**Clinical parameters at first sIgG4 measurement**								
ratio sIgG4	0.43 (0-1.61)	0.31 (0-0.85)	1.23 (1.01–1.61)	< 0.0001		1.16 (1.01–1.41)*	1.33 (1.18–1.61)	n.s.
Bilirubin (mg/dL)	1.18 (0.15–19.85)	1.21 (0.15–19.85)	0.96 (0.37–2.24)	n.s.		0.81 (0.37–1.36)	1.15 (0.43–2.24)	n.s.
ALT (U/L)	88 (11-1063)	93 (11-1063)	53 (15–160)	n.s.		39 (15–60)	72 (22–160)	n.s.
AST (U/L)	72 (14–819)	75 (14–819)	51 (25–150)	n.s.		53 (25–150)	49 (25–89)	n.s.
AP (U/L)	176 (35–741)	176 (35–741)	173 (49–433)	n.s.		136 (49–237)	222 (91–433)	n.s.
gGT (U/L)	166 (8-735)	167 (8-735)	158 (8-640)	n.s.		101 (8-261)	234 (57–640)	n.s.
**Clinical parameters at final sIgG4 measurement**								
ratio sIgG4	0.39 (0.01–2.20)	0.28 (0.01–0.92)	1.13 (0.67–2.2)	< 0.0001		0.83 (0.67-1.00)*	1.44 (1.19–2.20)	0.0007
Bilirubin (mg/dL)	1.03 (0.15–7.58)	1.04 (0.15–7.58)	0.94 (0.35 − 3.2)	n.s.		0.69 (0.35–1.32)	1.28 (0.46–3.24)	n.s.
ALT (U/L)	60 (5-705)	59 (9-705)	65 (5-314)	n.s.		34 (5–62)	107 (18–314)	n.s.
AST (U/L)	53 (14–455)	53 (14–455)	50 (18–182)	n.s.		32 (18–55)	73 (24–182)	n.s.
AP (U/L)	194 (47–902)	190 (47–800)	224 (58–902)	n.s.		113 (58–256)	371 (102–902)	0.0200
gGT (U/L)	127 (5-1007)	130 (5-1007)	109 (12–404)	n.s.		57 (12–164)	179 (27–404)	0.0180
Presence of IBD	79 (71.8%)	68 (70.8%)	11 (78.6%)	n.s.		6 (75.0%)	5 (83.3%)	n.s.
Presence of PSC-AIH variant-syndrome	24 (21.8%)	22 (22.9%)	2 (14.3%)	n.s.		1 (12.5%)	0 (0.0%)	n.s.
Presence of cirrhosis	19 (17.3%)	17 (17.7%)	2 (14.3%)	n.s.		1 (12.5%)	1 (16.7%)	n.s.
Liver transplantation	3 (2.7%)	3 (2.7%)	0 (0%)	n.s.		0 (0%)	0 (0%)	n.s.
Death during observation time	2 (1.8%)	2 (1.8%)	0 (0%)	n.s.		0 (0%)	0 (0%)	n.s.

Mean values (range) are presented for age and laboratory parameters

P1-values indicate differences between PSC-N and PSC-IgG4

P2-values indicate differences between PSC-IgG4norm and PSC-IgG4const

* indicate signifcant differences between first and final sIgG4 measurement

### sIgG4 levels during follow-up

sIgG4 levels were measured sequentially over a mean period of 3.5 years (range: 0.2–13.2) but at least at two different time points. When analyzing the sIgG4 levels at the first blood sampling, 96 PSC patients showed normal sIgG4 values (ratio ≤ 1.0; PSC-N) whereas sIgG4 was elevated in 14 individuals (ratio > 1.0; PSC-IgG4) demonstrating a sIgG4 elevation in 12.7% of our PSC cohort (Table 1).

**Fig. 1:**
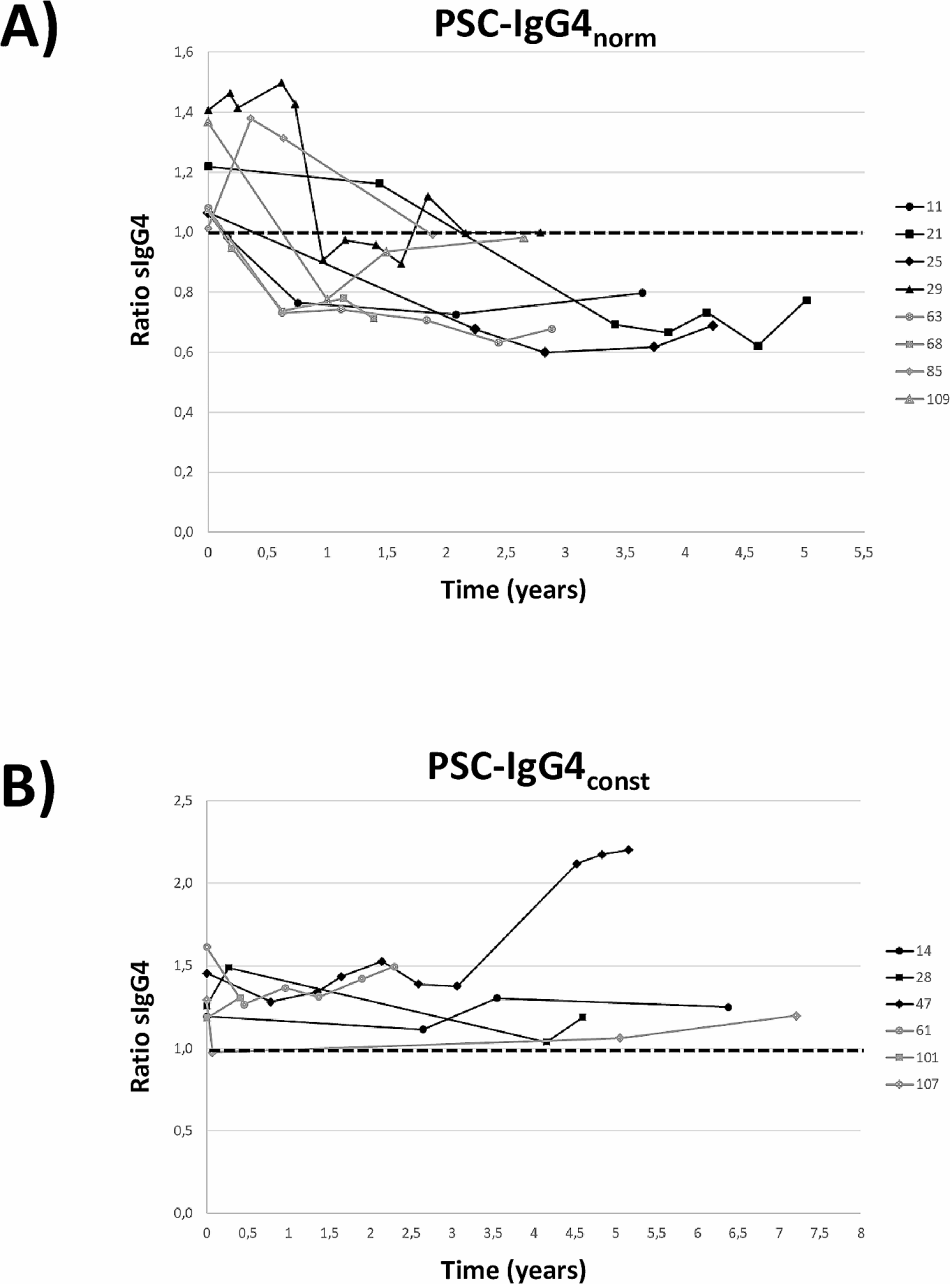
sIgG4 levels during follow-up in PSC-IgG4_norm_ and PSC-IgG4_const_ subgroups. During the observation time, ratios of sIgG4 normalized in eight patients (PSC-IgG4_norm_; Fig. 1**A**), whereas sIgG4 remained above the ULN in six patients (PSC-IgG4_const_; Fig. 1**B**). Although four of six patients (#28, #47, #61, #101) in the PSC-IgG4_const_ subgroup received immunosuppressive medication to treat IBD, sIgG4 levels stayed constantly above the ULN during the observation time. In the PSC-IgG4_norm_ subgroup, only two patients (#29, #85) received immunosuppressives. Dashed lines represent the ULN (ratio 1.0)

Considering the overall course of sIgG4 measurements, 96 of 110 (87.3%) PSC patients never had elevated sIgG4 levels during the observation time (PSC-N). In patients who showed an initial sIgG4 elevation (PSC-IgG4; *n* = 14), the values normalized in 57.1% (*n* = 8; PSC-IgG4_norm_) during follow-up measurements (Fig. 1A), whereas the values remained permanently elevated in 42.9% (*n* = 6; PSC-IgG4_const_) (Fig. 1B). In PSC-IgG4_norm_ only two of eight patients received immunosuppressive therapy (due to their IBD), although sIgG4 normalization was detected in all individuals. Likewise, sIgG4 levels remained above the ULN during the observation time in the PSC-IgG4_const_ subgroup although nearly all patients (4 of 6) received immunosuppressive therapy for IBD.

### Demographic and clinical characteristics in PSC-N versus PSC-IgG4 subgroups

The PSC-IgG4 subgroup consisted of significantly more male PSC patients as compared to PSC-N (Table 1; *p* = 0.008). However, age at PSC diagnosis, presence of IBD, high-grade biliary strictures, fibrosis using transient elastography detected by Fibroscan^®^, and cirrhosis as well as the Amsterdam Oxford, Mayo risk and MELD scores did not differ between the PSC-N and PSC-IgG4 subgroups (data not shown). Moreover, no differences in bilirubin, ALT, AST, AP, and γGT were observed between PSC-N and PSC-IgG4 patients (Table 1).

### Demographic and clinical characteristics in PSC-IgG4_norm_ versus PSC-IgG4_const_ subgroups

To characterize the PSC-IgG4 subgroup in more detail, we divided this group into patients with sIgG4 values that normalized during the observation period (PSC-IgG4_norm_; *n* = 8), and patients with sIgG4 ratios constantly at > 1.0 during the observation period (PSC-IgG4_const_; *n* = 6). Of note, the PSC-IgG4_const_ subgroup consisted of 100% male patients.

**Fig. 2:**
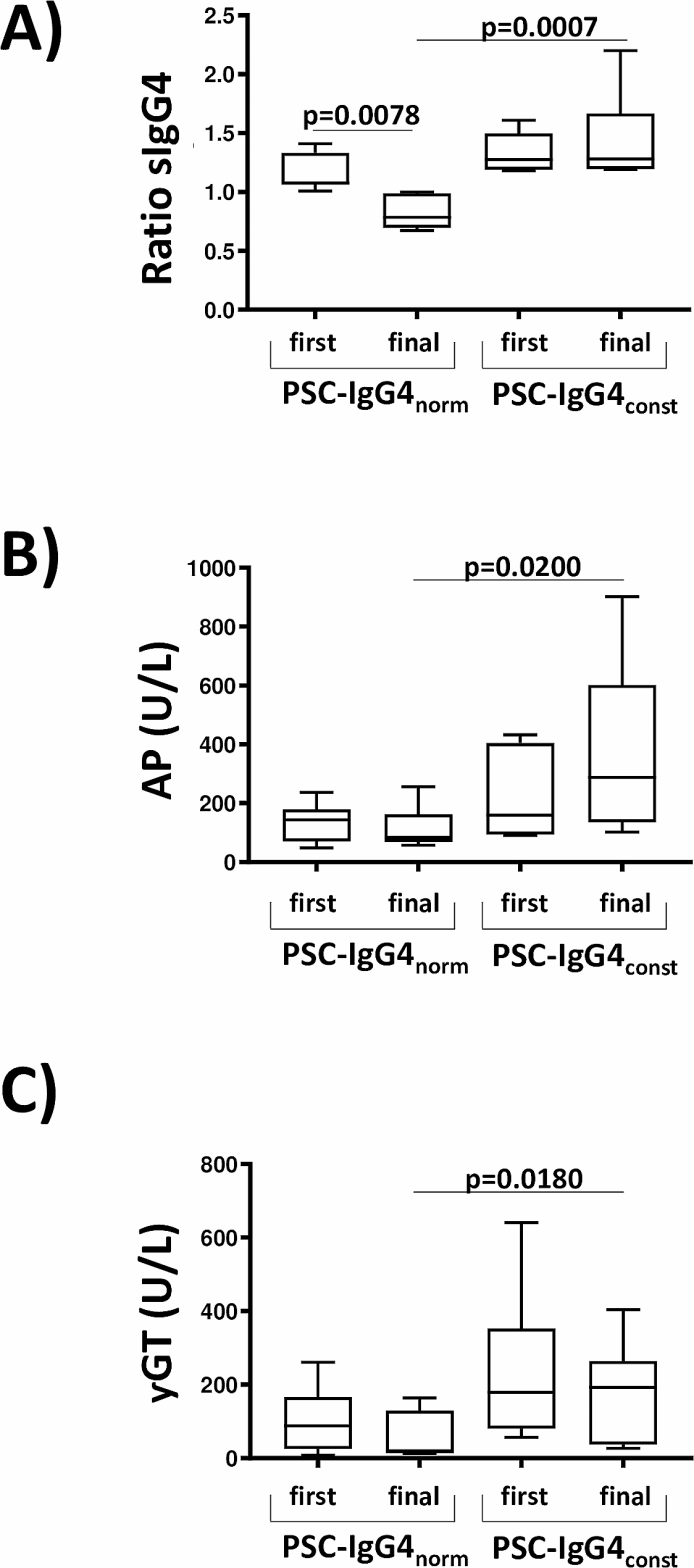
sIgG4, AP, and γGT in PSC-IgG4_norm_and PSC-IgG4_const_ subgroups. Clinical parameters of first blood sampling were compared to data of the final examination. Figure 2**A** shows that values of sIgG4 were significantly higher in PSC-IgG4_const_ patients than in PSC-IgG4_norm_ patients at final sIgG4 measurement. Serum values of AP and γGT were also higher in PSC-IgG4_const_ compared to PSC-IgG4_norm_ at final blood sampling (Fig. 2B and C). *P*-values refer to calculations using Wilcoxon matched-paired signed rank test and unpaired non-parametric Mann Whitney test for the comparisons marked by bars

To study differences in both subgroups, clinical parameters of first blood sampling were compared with data of the final examination. Values of sIgG4 were higher in PSC-IgG4_const_ patients in comparison to PSC-IgG4_norm_ patients at final sIgG4 measurement (Fig. 2A). Of note, serum values of AP and γGT were significantly higher in PSC-IgG4_const_ compared to PSC-IgG4_norm_ at final blood sampling (Fig. 2B and 2C).

Interestingly, mean age at PSC diagnosis was markedly lower in PSC-IgG4_const_ compared to PSC-IgG4_norm_ (Table 1; *p* = 0.0067). However, as demonstrated for the comparison of PSC-N versus PSC-IgG4, no differences in the presence of IBD, PSC-AIH variant-syndrome, high-grade biliary strictures, fibrosis grade measured by transient elastography Fibroscan^®^, cirrhosis, and Amsterdam Oxford Score were observed (data not shown).

## Discussion

In this monocentric retrospective study, we investigated the relevance of sequential measurements of sIgG4 regarding clinical parameters and outcome in order to accurately distinguish between the PSC phenotype with high sIgG4 and PSC with low sIgG4. Elevated sIgG4 levels in PSC were expected to be associated with higher cholestatic serum parameters and a shorter transplant-free survival time [5, 9, 10]. Our data demonstrated that 14 (12.7%) of these 110 PSC patients had elevated sIgG4 which is in agreement to the prevalence reported in the literature [5, 9, 14]. However, we could show that eight of these 14 patients exhibited a spontaneous normalisation of sIgG4 values (PSC-IgG4_norm_) during the observation period, whereas in six individuals, sIgG4 values continuously remained above ULN (PSC-IgG4_const)_. Our data therefore indicate that a single examination of sIgG4 for the identification of the PSC phenotype with high sIgG4 is not sufficient to assess this specific subgroup. Given that the observation time did not significantly differ between PSC-IgG4_norm_ and PSC-IgG4_const_ subgroup and was even slightly longer in the PSC-IgG4_const_ subgroup, our data suggest that a potential later decrease in sIgG4 levels was not overlooked. Cholestatic liver enzymes stayed mainly stable over time in the PSC-IgG4_norm_ subgroup, while AP and γGT levels were higher at the time of the final sIgG4 measurement in PSC-IgG4_const,_ which might indicate a more severe disease progression in PSC-IgG4_const_ subgroup.

The EASL CPG recommends a single determination of sIgG4 only at time of PSC diagnosis, in order to identify IAC [6]. However, it remains unclear if sIgG4 levels in PSC-IgG4 patients change over time, and may thus have a significance beyond an initial diagnostic approach at the time of diagnosis of sclerosing cholangitis. Our data demonstrate that sIgG4 levels remained stable only in a subgroup of PSC patients, which might represent the subgroup with aggravated risk for disease progression. However, this subgroup was not found in previous publications, potentially because data of these studies based on a single sIgG4 measurement.

Interestingly, the PSC-IgG4_const_ subgroup consisted only of male patients. Furthermore, we demonstrated that young age at diagnosis as well as serum levels of AP and γGT were associated with elevated sIgG4, but we failed to show a correlation between sIgG4 and transplant free survival or risk models for disease severity in our PSC cohort. Although several studies have demonstrated a more progressive PSC in patients with high sIgG4 values [5, 9, 10], current data on the role of sIgG4 remains inconclusive [8].

Bjornsson et al. reported that corticosteroid-therapy ameliorates cholestatic serum features in a small group of PSC patients with elevated IgG4 [10]. The EASL CPG does not suggest to use corticosteroids in PSC patients with mildly elevated sIgG4 (< 2x ULN) [6], however, steroid therapy is recommended as standard treatment for IAC. Our data indicate that a single sIgG4 measurement cannot reliably distinguish the PSC-IgG4 group with constant sIgG4 elevation (PSC-IgG4_const_) that might be at risk for PSC disease progression.

In the subgroup of PSC-IgG4_norm_, sIgG4 values normalized independent of immunosuppressive medication during the observation time. In the PSC-IgG4_const_ subgroup, sIgG4 remained elevated, although nearly all patients received immunosuppressive medication for their IBD. Therefore, an indication for immunosuppressive treatment for the PSC-IgG4 subgroup remains elusive, especially since only a small group had constant sIgG4 elevation and all of these patients received various immunosuppressive medications for a different indication (IBD). The identification of a PSC-IgG4_const_ subgroup seems to be more relevant in a prognostic prospective rather than a therapeutic approach. Our data do not allow to draw a therapeutic conclusion for the IgG4_const_ subgroup. Limitations of this study include the single center approach and the small number of patients. Therefore, findings should be confirmed with a larger number of patients. Additionally, we only present a mean follow up of 4.4 years.

In conclusion, this is the first study reporting sequential sIgG4 measurements in a well-characterized European PSC cohort. Our data show that normalization of sIgG4 occurred in the majority of PSC patients and that sequential determinations of sIgG4 levels allow to accurately distinguish between the PSC phenotype with high sIgG4 and PSC with low sIgG4.

### Electronic supplementary material

Below is the link to the electronic supplementary material.

Supplementary Material 1

## Data Availability

The datasets used and/or analyzed during the current study are available from the corresponding author (SK) on reasonable request.
