# Supplementary figures and images for: Only repeatedly elevated IgG4 levels in primary sclerosing cholangitis may distinguish a particular patient phenotype
Source: BMC Gastroenterol. 2024 Aug 5;24:248. doi: 10.1186/s12876-024-03343-3 (PMC11301849; doi:10.1186/s12876-024-03343-3)

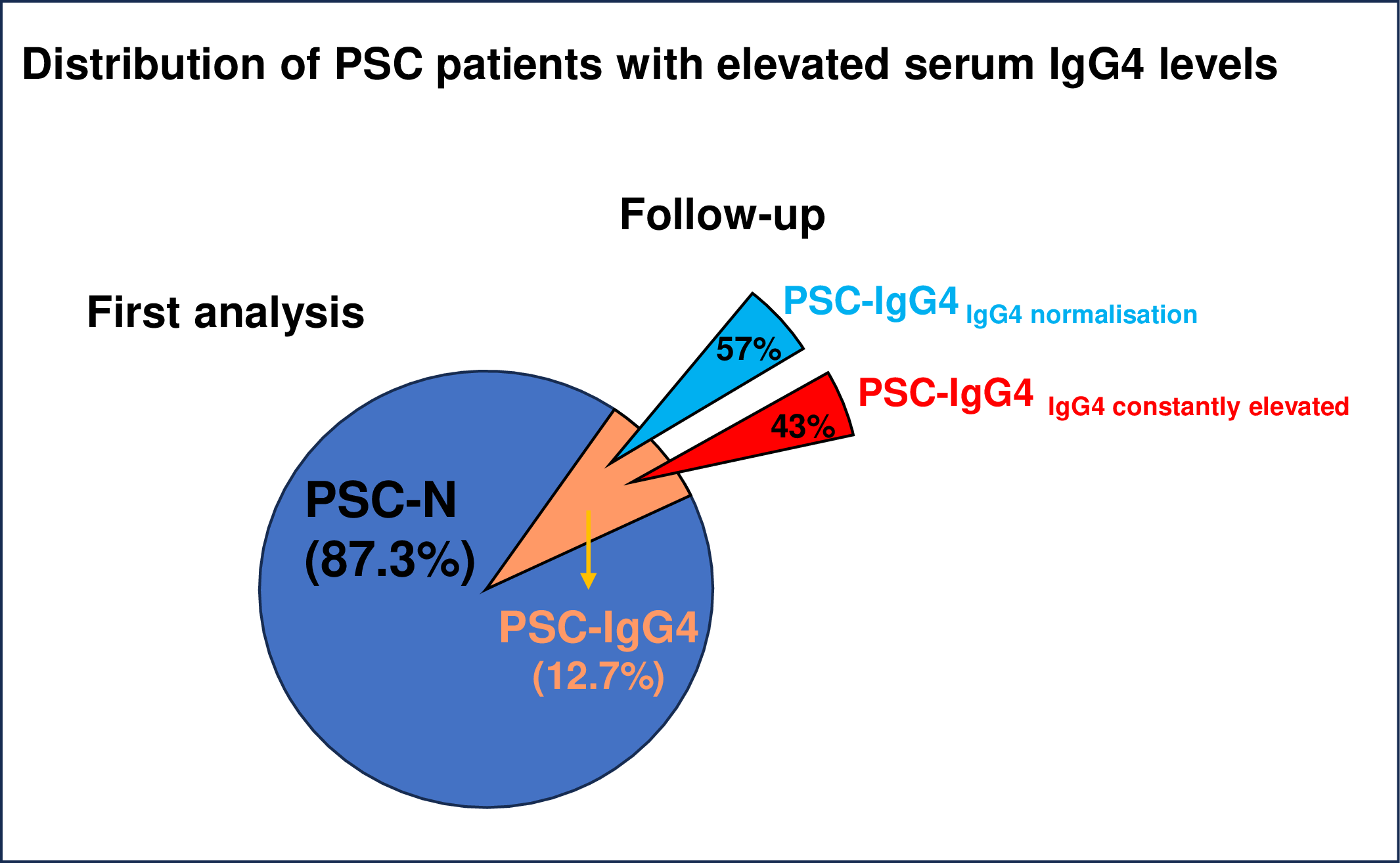

Supplement: Supplementary file 1 — Supplementary Material 1 [file 12876_2024_3343_MOESM1_ESM.tif]
